# X-ray sensitivity of fibroblasts from patients with hereditary retinoblastoma and their families.

**DOI:** 10.1038/bjc.1987.125

**Published:** 1987-06

**Authors:** J. V. Pledger, A. W. Craft, K. Bartlett, D. R. Long

## Abstract

The in vitro response to X-irradiation of cultured human fibroblasts was studied using a colony forming assay. A comprehensive reference range was established, giving a median D0 value of 98.5 cGy with an interquartile range of 86.5-110.5 cGy. Cells from 3 retinoblastoma family pedigrees were studied and the cell survival after exposure to X-rays was compared between affected (11 samples) and unaffected (26 samples) family members. No significant differences in response to ionising radiation were found between the controls, the affected and the unaffected members of the 3 families. The affected members had a median D0 of 97.5 cGy (interquartile range 87.5-107.5 cGy) and the unaffected members had a median D0 of 102 cGy (interquartile range 93-111 cGy). Thus radiosensitivity is not a useful marker for the detection of the retinoblastoma gene.


Br. J. Cancer (1987), 55, 617–621 © The Macmillan Press Ltd., 1987

### X-ray sensitivity of fibroblasts from patients with hereditary retinoblastoma and their families

J.V. Pledger, A.W. Craft, K. Bartlett & D.R. Long

*Department of Child Health, The Medical School, University of Newcastle Upon Tyne, NE2 4HH, UK.*

**Summary** The *in vitro* response to X-irradiation of cultured human fibroblasts was studied using a colony forming assay. A comprehensive reference range was established, giving a median $D_0$ value of 98.5 cGy with an interquartile range of 86.5–110.5 cGy. Cells from 3 retinoblastoma family pedigrees were studied and the cell survival after exposure to X-rays was compared between affected (11 samples) and unaffected (26 samples) family members.

No significant differences in response to ionising radiation were found between the controls, the affected and the unaffected members of the 3 families. The affected members had a median $D_0$ of 97.5 cGy (interquartile range 87.5–107.5 cGy) and the unaffected members had a median $D_0$ of 102 cGy (interquartile range 93–111 cGy).

Thus radiosensitivity is not a useful marker for the detection of the retinoblastoma gene.

Retinoblastoma is a malignant eye tumour that can occur both sporadically and in genetically predisposed individuals. Children with hereditary retinoblastoma have an increased risk of developing other tumours later in life, particularly osteosarcomas, at sites distant from the eye. They also have a high incidence of radiation induced tumours around the eye. Whether or not these patients or their cultured cells are usually sensitive to the effects of x-irradiation has been a matter of controversy with some research groups showing an increased sensitivity (Weichselbaum *et al.*, 1980, Arlett & Harcourt, 1980, Weichselbaum *et al.*, 1985) whilst others have shown it to be normal (Kossakowska *et al.*, 1982; Zampetti *et al.*, 1981, Ejima *et al.*, 1982).

In order to attempt to clarify the question we have studied the radiosensitivity of fibroblasts from affected and unaffected members of three retinoblastoma families as well as establishing a reference range on age and sex matched normal subjects.

### Patients and methods

Skin biopsies were obtained at surgical operation from patients without malignant disease to provide a reference range of radiosensitivities of human fibroblast cell lines. Sixteen cell lines were assayed, nine females and seven males with an age range of 2 months to 56 years, corresponding to that of the retinoblastoma family members. An ataxia-telangiectasia line was obtained from Dr R. Cox at the Radiobiology Unit, Harwell and was used as a positive radiosensitive control.

Three of the four families in the Northern region, in which at least two members are affected by retinoblastoma, were available for study and their pedigrees are shown in Figures 1 and 2. The family members who are affected by retinoblastoma are represented by blocked symbols and those who have tumours which have regressed by hatched symbols.

Three members of family 1 died of retinoblastoma (II-1, III-5 and III-4), II-4 and I-1 died of carcinoma of the bladder and II-7 died of carcinoma of the cervix. III-12, who was an affected member, died of pneumonia. II-8 had bilateral retinoblastoma and later developed osteogenic sarcoma of the femur and died. III-9 is apparently normal. III-10 had unilateral retinoblastoma as a baby and also developed osteogenic sarcoma of the femur at the age of 11 and remains well following treatment at the age of 15 years.

Correspondence: A.W. Craft.
Received 10 October 1986; and in revised form 19 January 1987.

Families 2 and 3 each have two affected members. All members of the families who were still alive took part in the study except for III-1, III-3 and III-16 in family 1 and II-2 in family 3. Skin biopsies were taken from affected retinoblastoma patients (11) and all available unaffected members (26) of their families.

Fibroblasts were grown in Eagles minimal essential medium supplemented with 10% foetal calf serum (Gibco Ltd), 0.2 mg of L-glutamine, 0.5 mg of streptomycin and 500 units of penicillin. They were maintained at 37°C in a 95% air/5% CO atmosphere during culture and then stored in liquid nitrogen.

X-ray survival experiments were carried out using cells which had just reached confluence. They were removed from stock culture with 0.25% trypsin in calcium and magnesium free Hanks balanced salt solution. To prepare a 'feeder layer' a cell suspension of $2.5 \times 10^5$ cells ml$^{-1}$ was exposed to 30 Gy of X-rays and 0.2 ml of this suspension was dispensed onto each plate.

Appropriate numbers of viable cells were seeded into triplicate 9 cm petri dishes and incubated overnight. The medium was then removed and the dishes exposed to X-ray doses of 50, 100, 150, 200 and 300 cGy. All irradiation was carried out on a Marconi deep X-ray therapy machine using a 240 KV source, a 2 mm Al filter and an HVT of 1.1 mm Cu. Fresh medium was added and the dishes incubated for 14 days, when the cells were stained with Azur A. Colonies of more than 50 cells were counted as surviving. $D_0$ values were determined by linear regression analysis for each cell line. The results from each group of cell lines were expressed using a median value with its interquartile range. The results of the different groups were compared using Kolmogorov–Smirnov two-sample statistical analysis.

### Results

The results of the radiation sensitivity studies are summarised in Table I and the individual results are shown in Table II. Median and interquartile range is quoted where the group is large enough otherwise the mean and range is given. There was no significant difference between the control fibroblasts and either the affected or unaffected members from family 1. There were insufficient numbers in either families 2 or 3 to make valid statistical comparisons. The data was therefore combined into affected (11) and unaffected (26) and these combined results are summarised in Table III and Figure 3. Once again there are no significant differences between the groups. The cell survival curve for the AT cell line is also shown in Figure 3.

**Figure 1** Pedigree of family 1.

**Figure 2** Pedigree of families 2 and 3.

**Table I** Summary of radiation sensitivity studies from 3 families

| | *Group* | *n* | *Median $D_0$ (cGy)* | *Interquartile range* | *Passage number* | *Plating efficiency* |
|---|---|---|---|---|---|---|
| | Control | 16 | 98.5 | 86.5–110.5 | 3–11 | 14–79 |
| Family 1 | Affected | 7 | 101.6 | 93.6–109.6 | 4–13 | 7–62 |
| | Unaffected | 14 | 98.5 | 86.8–110.3 | 4–15 | 6–63 |
| | | | *Mean $D_0$ (cGy)* | *Range* | | |
| Family 2 | Affected | 2 | 91 | 87.0– 95 | 5–7 | 17–63 |
| | Unaffected | 7 | 103.5 | 91.0–120 | 5–17 | 13–67 |
| Family 3 | Affected | 2 | 93 | 91.0– 95 | 4–14 | 16–38 |
| | Unaffected | 5 | 107.9 | 101.0–113 | 5–9 | 18–60 |
| | AT cell line | 1 | 36.0 | | | 8.0 |

**Table II** Results of individual experiments in the 3 families and normals

| *Normal* | | | | | *Unaffected family members* | | | | | *Affected family members* | | | | |
|---|---|---|---|---|---|---|---|---|---|---|---|---|---|---|
| *Control number* | *Passage number* | *Plating efficiency* (%) | $D_0$/ *cGys* | *Mean* $D_0$ | *Pedigree number* | *Passage number* | *Plating efficiency* (%) | $D_0$/ *cGys* | *Mean* $D_0$ | *Pedigree number* | *Passage number* | *Plating efficiency* (%) | $D_0$/ *cGys* | *Mean* $D_0$ |
| | | | | | *Family 1* | | | | | *Family 1* | | | | |
| 1 | 6 | 18.6 | 66 | 66 | II 2 | 8 | 48.2 | 120 | 112 | II 6 | 4 | 36 | 103 | 97.5 |
| 2 | 6 | 79.3 | 98 | | | 8 | 41.2 | 104 | | | 4 | 42.4 | 92 | |
| | 6 | 56.3 | 115 | 105.7 | II 3 | 4 | 34.2 | 95 | 98.5 | II 12 | 4 | 23 | 77 | 76.6 |
| | 10 | 54.8 | 104 | | | 6 | 16 | 102 | | | 13 | 8.3 | 77 | |
| 3 | 4 | 52.3 | 100 | 100 | II 5 | 5 | 44.2 | 91 | 94 | | 13 | 7.5 | 76 | |
| 4 | 7 | 35.6 | 100 | 100 | | 6 | 38.2 | 92 | | II 14 | 6 | 38.6 | 114 | 111 |
| 5 | 7 | 14.4 | 85 | 77.5 | II 8 | 9 | 20.8 | 99 | | | 6 | 35.6 | 108 | |
| | 7 | 23.7 | 70 | | | 8 | 37.7 | 94 | 98.5 | III 4 | 8 | 51.2 | 133 | |
| 6 | 3 | 31.6 | 80 | 80 | II 9 | 8 | 34.1 | 103 | | | 10 | 56.8 | 146 | 136.6 |
| 7 | 6 | 64.8 | 104 | 104 | | 4 | 30.6 | 78 | | | 11 | 71.8 | 131 | |
| 8 | 6 | 54 | 80 | 80.5 | | 5 | 34 | 75 | 76.3 | III 10 | 4 | 44.5 | 103 | |
| | 6 | 47 | 81 | 80.5 | II 10 | 8 | 21.7 | 76 | | | 9 | 61.7 | 95 | 101.6 |
| 9 | 6 | 27 | 104 | 105 | II 11 | 13 | 8.6 | 97 | 97 | | 10 | 46.2 | 107 | |
| | 10 | 30 | 106 | | | 5 | 59.5 | 123 | 123 | III 11 | 4 | 40.3 | 101 | 101.5 |
| 10 | 10 | 25.3 | 131 | 129.5 | II 13 | 6 | 55.2 | 123 | | | 4 | 31 | 102 | |
| | 11 | 39.5 | 128 | | | 14 | 5.6 | 71 | 68.5 | III 13 | 7 | 50 | 106 | 115.5 |
| 11 | 7 | 45.8 | 92 | 91.5 | II 15 | 15 | 6.4 | 66 | | | 7 | 36.9 | 125 | |
| | 7 | 41.7 | 91 | | | 6 | 15.3 | 81 | 88.5 | | | | | |
| 12 | 7 | 47.4 | 109 | 97 | III 2 | 10 | 25.3 | 96 | | | | | | |
| | 7 | 29.8 | 85 | | | 6 | 28.6 | 90 | 85.5 | | | | | |
| 13 | 5 | 51.5 | 83 | 82.5 | III 6 | 6 | 29.2 | 81 | | | | | | |
| | 5 | 35.7 | 82 | | | 5 | 61.7 | 118 | 114.5 | | | | | |
| 14 | 5 | 59.3 | 87 | 92 | III 7 | 5 | 56 | 111 | | | | | | |
| | 5 | 50.1 | 97 | | | 6 | 55.3 | 102 | 103 | | | | | |
| 15 | 4 | 72.4 | 129 | 121.5 | III 9 | 7 | 62.8 | 104 | | | | | | |
| | 4 | 65.4 | 114 | | | 7 | 43 | 121 | 118 | | | | | |
| 16 | 7 | 70.2 | 131 | 126.5 | III 10 | 7 | 35 | 115 | | | | | | |
| | 7 | 54.6 | 122 | | | 10 | 18.2 | 99 | 99 | | | | | |
| | | | | | *Family 2* | | | | | *Family 2* | | | | |
| | | | | | I 1 | 6 | 28.6 | 120 | 114 | II 1 | 7 | 62.8 | 92 | 95 |
| | | | | | | 6 | 27.4 | 108 | | | 7 | 46.4 | 98 | |
| | | | | | I 2 | 7 | 16.5 | 98 | | III 1 | 5 | 18.4 | 80 | 97 |
| | | | | | | 8 | 44.9 | 91 | 94 | | 5 | 16.9 | 94 | |
| | | | | | | 8 | 36.1 | 93 | | | | | | |
| | | | | | II 2 | 5 | 36.5 | 107 | 107 | | | | | |
| | | | | | II 3 | 6 | 66.6 | 95 | 96 | | | | | |
| | | | | | | 7 | 53.3 | 97 | | | | | | |
| | | | | | II 4 | 9 | 32.4 | 101 | 104 | | | | | |
| | | | | | | 17 | 19.8 | 107 | | | | | | |
| | | | | | II 5 | 12 | 19.6 | 91 | 94 | | | | | |
| | | | | | | 13 | 12.7 | 97 | | | | | | |
| | | | | | II 6 | 5 | 64 | 113 | 115.5 | | | | | |
| | | | | | | 5 | 53.8 | 118 | | | | | | |
| | | | | | *Family 3* | | | | | *Family 3* | | | | |
| | | | | | I 1 | 5 | 42.9 | 115 | 112.5 | II 1 | 4 | 31.4 | 92 | 95 |
| | | | | | | 5 | 46.6 | 110 | | | 4 | 16.4 | 98 | |
| | | | | | I 2 | 7 | 18.6 | 101 | 101 | III 1 | 10 | 29.2 | 100 | 91 |
| | | | | | II 3 | 6 | 41.5 | 105 | 111.5 | | 14 | 38.2 | 82 | |
| | | | | | | 6 | 31.2 | 118 | | | | | | |
| | | | | | II 4 | 7 | 26.2 | 104 | 104 | | | | | |
| | | | | | III 2 | 9 | 50.8 | 110 | 110.5 | | | | | |
| | | | | | | 9 | 59.7 | 111 | | | | | | |

**Table III** Combined results of all 3 families

| *Experimental group* | *Median* $D_0$ *value/cGy* | *Interquartile range/cGy* | *Samples in group* |
|---|---|---|---|
| Control | 98.5 | 86.5–110.5 | 16 |
| Affected | 97.5 | 87.5–107.5 | 11 |
| Unaffected | 102.0 | 93.0–111.0 | 25 |

Two other fibroblast cell lines from patients with retinoblastoma were assayed that did not belong to the family pedigrees. The first was from a patient who has a balanced 13q14 translocation and the second was from a patient with a chromosomal deletion between 13q14 and 13q22. Neither of these lines showed any abnormality with respect to their radiosensitive responses, the $D_0$ values being 95 cGy and 110 cGy respectively.

### Discussion

The methods of determining normal ranges in other studies have varied. The details are not always included in the literature but the available data from the major studies are shown in Table IV. The methods used to obtain control ranges vary widely. Weichselbaum *et al.* (1980), used 6 normal lines and assayed them repeatedly. The resulting range was thus limited, being dependent on the number of cell lines and not on the number of assays. Arlett and Harcourt (1980), studied only 2 normal lines and then each time an experimental line was assayed, one of the 2 normals was assayed in parallel. If the experimental line radiosensitivity fell within the range of either of the 2 normals then it too was considered to have a normal response to ionising radiation. By this means a control range of 97–190 cGy was obtained. Cox and Masson (1980), whilst

**Figure 3** Cell survival curves for unaffected family members (A), normal subjects (B), affected family members (C), known A-T cell line (D).

studying A–T cell lines, reported a reference range from 20 normal cell lines assayed in duplicate. They obtained a range of 98–160 cGy with a distribution skewed towards the lower end.

The control range in the present study is at the lower end of that reported by Cox and Masson (1980) and Arlett and Harcourt (1980) but considerably below that of Weichselbaum *et al.* (1980). The latter, however, is very narrow when compared with other control ranges. It is clearly necessary for each laboratory to establish its own control ranges as assay conditions between one laboratory and the next will vary, particularly with respect to culture conditions and the establishment of the cell line.

An additional criticism of the method of Weichselbaum *et al.* (1980) is the fact that a feeder layer was not used. This means that the plating efficiency was relatively low, ranging from 0.4–44%. Some of the same cell lines, however, were also assayed by Arlett and Harcourt (1980) using feeder layers. These workers obtained higher plating efficiencies and $D_0$ values which were in agreement with the earlier study. Many previous workers have used a wider range of radiation dose, occasionally 0–1000 cGy. In the present study a dose range of 0–300 cGy was used. This was partly due to restrictions imposed by the apparatus used but also because it was considered that as early passage diploid fibroblasts were being assayed, one log of survival would be adequate.

In all studies of response to ionising radiation using cell cloning assays, A–T cell lines are used as positive reference. The plating efficiency of A–T lines is generally recognised as being low and even with a feeder layer still only reached 8.0% in the present study. The $D_0$ was 36 cGy which is well below the lower end of the control range.

Retinoblastoma is known to be a hereditary form of cancer and here three pedigrees are studied in detail. Family 1 is large enough for the results to be compared separately to the controls. When divided into affected family members (7) and unaffected family members (14) and compared to the controls, no difference in median $D_0$ or interquartile range could be detected.

Families 2 and 3 are relatively small and their results were combined with family 1 for comparison with controls and again there were no significant differences.

We conclude therefore from this study that no difference can be shown between controls, affected and unaffected members of families in which retinoblastoma is known to be inherited. Thus if the tendency to malignancy, either retinoblastoma or secondary primary tumours, is related to an inability to repair damaged DNA, it is unlikely to be the

**Table IV** Previous studies on radiosensitivity of skin fibroblasts from retinoblastoma patients

| | *No. cell lines* | *No. assays* | *$D_0$ mean (cGy)* | *Range* | *Type of Rb* | *Number of patients* | *Radio-sensitive responses* |
|---|---|---|---|---|---|---|---|
| Weichselbaum *et al.* | 6 | 18 | 147 | 140–152 | Hereditary[a] | 6 | + |
| (1980) | | | | | Sporadic[b] | 7 | – |
| | | | | | D-Deletion[c] | 1 | + |
| | | | | | D-Deletion | 1 | – |
| Arlett & Harcourt | 2 | | see | 97–180 | Hereditary | 3 | + |
| (1980) | | | text | | Mother of | | |
| | | | | | Bilateral | 1 | + |
| | | | | | Sporadic | 1 | – |
| | | | | | D-Deletion[c] | | |
| Kossakowska *et al.* | 8 | 16 | 118 | not | Hereditary | 10 | – |
| (1982) | | | | given | Siblings | 5 | – |
| Cox & Masson | 20 | 40 | see | 98–160 | Not given | | |
| (1980) | | | text | | | | |
| Zampetti-Bosellar & Scott | 4 | — | 86 | 78–101 | D-Deletion | 1 | – |
| (1981) | | | | | | | |
| Present study | 16 | 32 | 98.5 | 87–111 | Hereditary | 11 | – |
| | | | | | Family members | 26 | – |
| Nove *et al.* | | | | | Hereditary[a] | 5 | + |
| (1979) | | | | | Hereditary | | |
| | | | | | Sporadic[b] | | – |
| Gallie | | | | | Hereditary | 8 | – |
| (1980) | | | | | Sporadic | 2 | – |
| | | | | | Siblings | 3 | – |
| Ejima *et al.* | | | | | Hereditary | 4 | – |
| (1982) | | | | | Sporadic | 10 | – |

Response + = radiosensitive; – = normal. [a]same cell line. [b]same cell line. [c]same cell line.

type of damage induced by ionising radiation that is involved.

These results disagree with the findings of Weichselbaum *et al.* (1980), Nove *et al.* (1979) and Arlett and Harcourt (1980). These groups all showed that hereditary retinoblastoma strains were sensitive and sporadic strains were not. They also showed that some D-deletion retinoblastoma lines showed a degree of sensitivity. The other groups whose results are summarised in Table 4 showed no sensitivity of fibroblasts to ionising radiation, Gallie (1980), Kossakowska *et al.* (1982) and Ejima *et al.* (1982). The results in the present study support the data of the latter groups.

In a number of other reports, retinoblastoma lines have been included as part of wider based studies, and these fibroblasts are considered to have a normal survival after exposure to ionising radiation. These include Zampetti-Bosseler and Scott (1981), Barfknecht and Little (1982) and Fujiwara *et al.* (1981). Harnden *et al.* (1980) and Cox and Masson (1980), while mainly investigating A–T have classified retinoblastoma as having normal radiosensitivity response.

Not all laboratories use identical methodologies, so it is perhaps not surprising that when the same cell lines have been studied in different laboratories, disparate results have been obtained. Nove *et al.* (1979) working in the same laboratory as Weichselbaum reinvestigated 11 cell lines previously assayed by him and the results were in agreement. Arlett and Harcourt (1980) assayed a D-deletion strain in common with Weichselbaum and confirmed that it was sensitive. This same strain however, was assayed by Zampetti-Bosselar and Scott (1981) and was classified as normal, although the reference range in this paper was lower than that of Weichselbaum and just within the lower end of that found by Arlett and Harcourt. Two of Weichselbaum's cell lines were assayed by Kossakowska *et al.* (1982), one showing a different and the other a similar reponse. Arlett and Priestly (1983) investigated the repair of potentially lethal damage (RPLD) on two retinoblastoma cell strains. They showed that the strain GM1142, which is one of the major controversial cell lines, continued to show radiation hypersensitivity and was defective in RPLD. However a cell strain from a familial retinoblastoma showed defective RPLD but normal radiosensitivity. They suggest that some of the discrepancy in experiments such as these may be implicit in experimental designs.

The ideal method of establishing a comprehensive reference range would be for an experimental cell line to be assayed with an age and sex matched control cell line simultaneously. This, however, due to the technical limitations of the assay method and the problems associated with obtaining such specific surgical specimens, is impractical. The method used in this study was the most comprehensive available for the determination of a reference range to cover the spread of age groups concerned.

Several D-deletion retinoblastoma cell strains have previously been studied with varying results. This may be because that if a gene controlling radiosensitivity should map close to the retinoblastoma gene on chromosome 13, then the size of the deletion might be the determining factor in whether a particular cell line with a deletion between 13q14 and 13q22 was assayed and found to be normal. Thus the gene controlling radiosensitivity, should it exist, is not likely to map on this region of chromosome 13. In the present study a cell line from a patient who has retinoblastoma and demonstrated a translocation involving 13q14 was assayed. This cell line exhibits no actual loss of chromosomal material and the radiosensitive response was found to be normal. A–T cells, whose radiosensitivity is reflected at the cellular level, are more sensitive to the lethal effects of ionising radiation than normal cells but there is no evidence of involvement of chromosome 13.

We conclude that no abnormal sensitivity to low dose ionising radiation can be detected using a colony forming assay in retinoblastoma families.

We thank the Department of Medical Physics, Royal Victoria Infirmary for assistance with the irradiation of the cells and the North of England Children's Cancer Research Fund and the Leukaemia Research Fund for financial support.

**References**

ARLETT, C.F. & HARCOURT, S.A. (1980). Survey of radiosensitivity in a variety of human cell strains. *Cancer Res.*, **40**, 926.

ARLETT, C.F. & PRIESTLY, (1983). Defective recovery from potentially lethal damage in some human fibroblast cell strains. *Int. J. Radiat. Biol.*, **43**, 157.

BARFKNECHT, T.R. & LITTLE, J.B. (1982). Survival of hereditary retinoblastoma fibroblasts after treatment with DNA damaging chemicals. *Mutation Res.*, **105**, 189.

COX, R. & MASSON, W.K. (1980). Radiosensitivity in cultured human fibroblasts. *Int. J. Radiat. Biol.*, **38**, 575.

EJIMA, Y., SASAKI, M.S., UTSUMI, H., KANEKO, A. & TANOOKA, H. (1982). Radiosensitivity of fibroblasts from patients with retinoblastoma and chromosome-13 anomalies. *Mutation Res.*, **103**, 177.

FUJIWARA, Y., MIYAZAKI, N., KANO, Y., TAKAHASHI, T. & KANEKO, A. (1981). X-ray, UV and chemical mutagen sensitivities of skin fibroblasts from patients with familial and chromosome 13q-retinoblastomas. *J. Radiat. Res.* (Tokyo), **22**, 472.

GALLIE, B.L. (1980). Gene carrier detection in retinoblastoma. *Opthalmology*, **87**, 591.

HARNDEN, D.G., EDWARDS, M., FEATHERSTONE, T., MORTEN, J., MORGAN, R. & TAYLOR, A.M.R. (1980). Studies on cells from patients who are cancer prone and who may be radiosensitive. In *Genetic and Environmental Factors in Experimental and Human Cancer*, Gelboin H.V. (ed) p. 231. Jap. Sci. Soc. Press.

KOSSAKOWSKA, A.E., GALLIE, B.L. & PHILLIPS, R.A. (1982). Fibroblasts from retinoblastoma patients; enhanced growth in fetal calf serum and a normal response to ionising radiation. *J. Cell Physiol.*, **111**, 15.

NOVE, J., WEICHSELBAUM, R.R., NICHOLS, W.W., ALBERT, D.M. & LITTLE, J. (1979). *In vito* studies of fibroblasts from patients with retinoblastoma. *Int. Ophthalmol Clin.*, **20**, 211.

WEICHSELBAUM, R.R., NOVE, J. & LITTLE, J.B. (1980). X-ray sensitivity of fifty-three human diploid fibroblast cell strains from patients with characterized genetic disorders. *Cancer Res.*, **40**, 920.

WEICHSELBAUM, R.R., TOMLINSON, K. & LITTLE, J.B. (1985). Repair of potentially lethal X-ray damage in fibroblasts derived from patients with hereditary and D-deletion retinoblastoma. *Int. J. Radiat. Biol.*, **47**, 445.

ZAMPETTI-BOSSELER, F. & SCOTT, D. (1981). Cell death, chromosome damage and mitotic delay in normal human, ataxia-telangiectasia and retinoblastoma fibroblasts after X-irradiation. *Int. J. Radiat. Biol.*, **39**, 547.


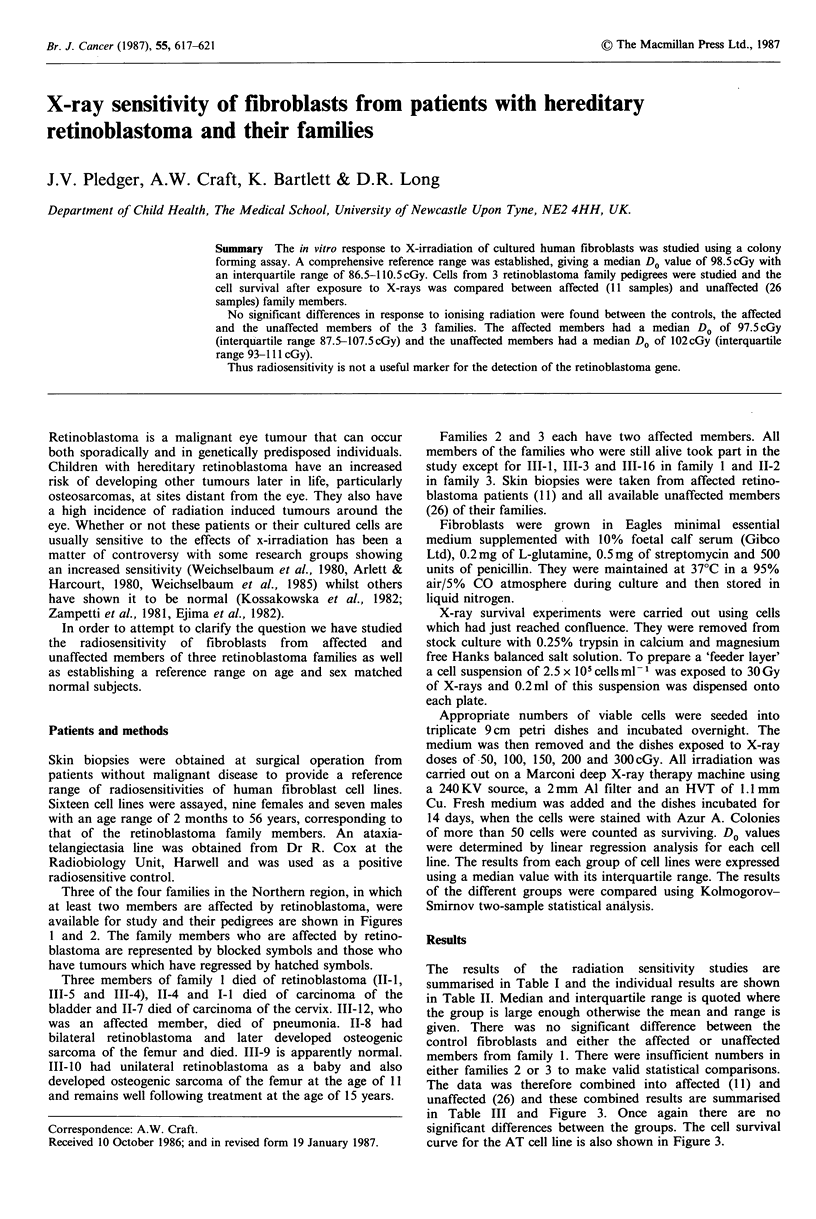

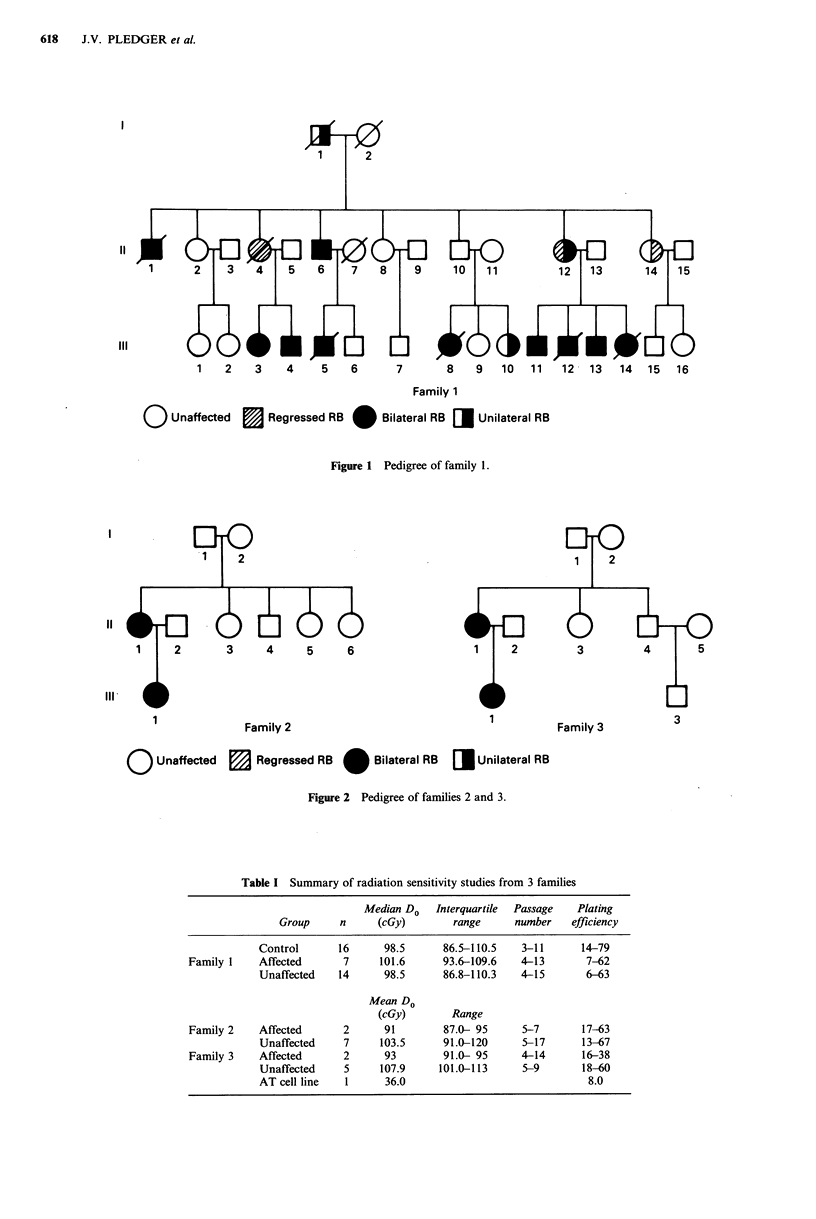

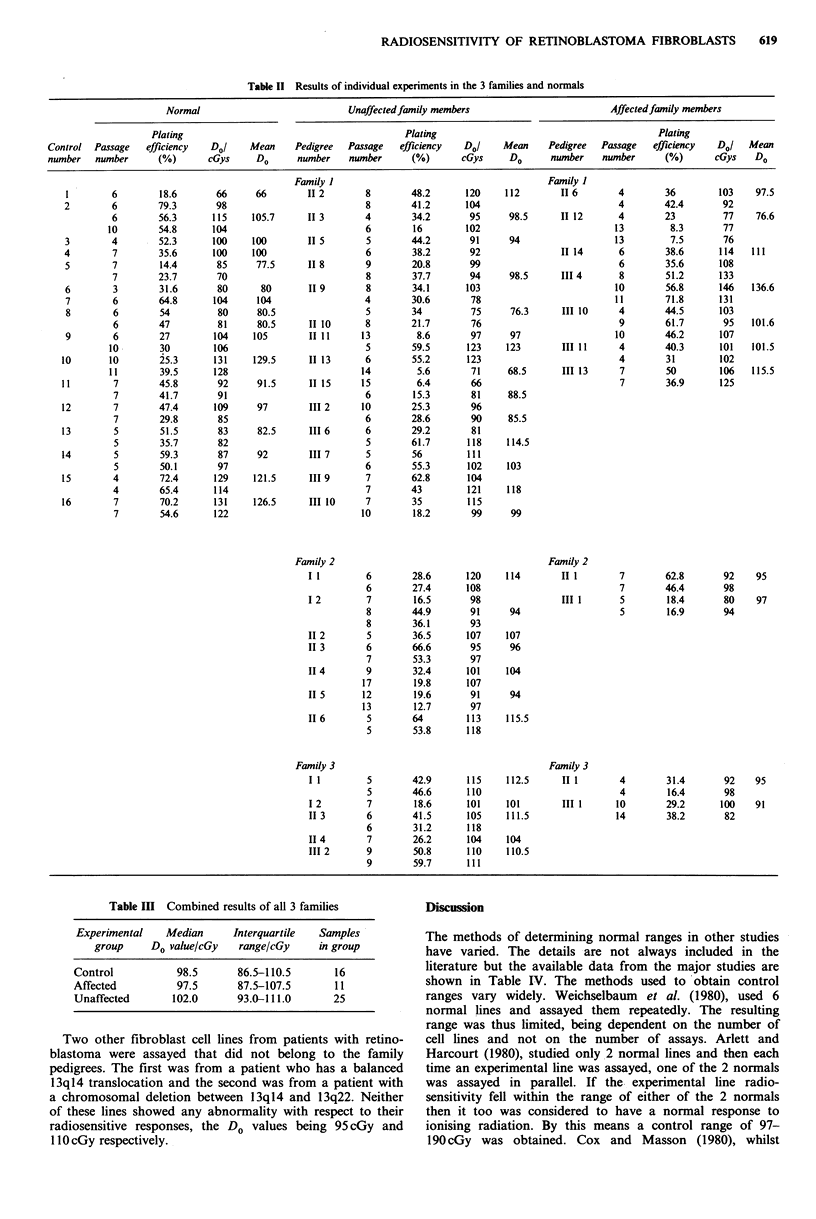

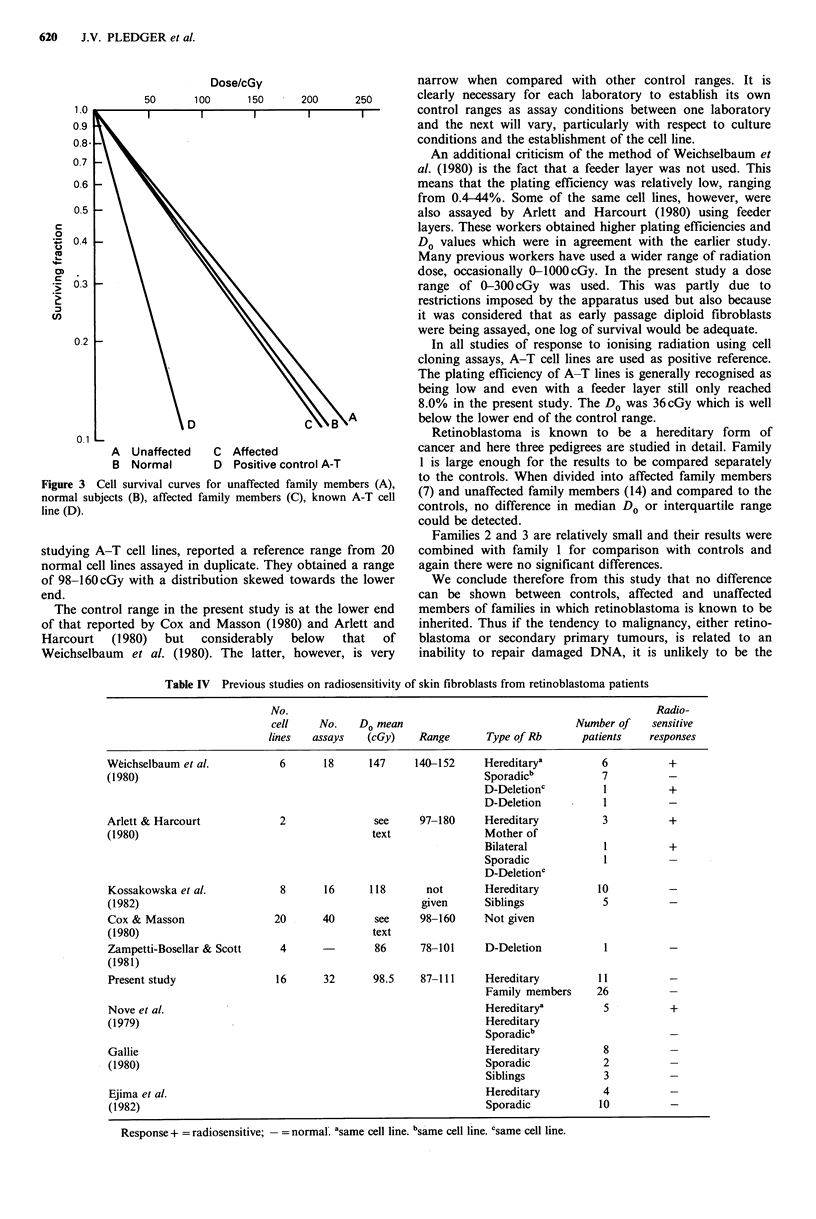

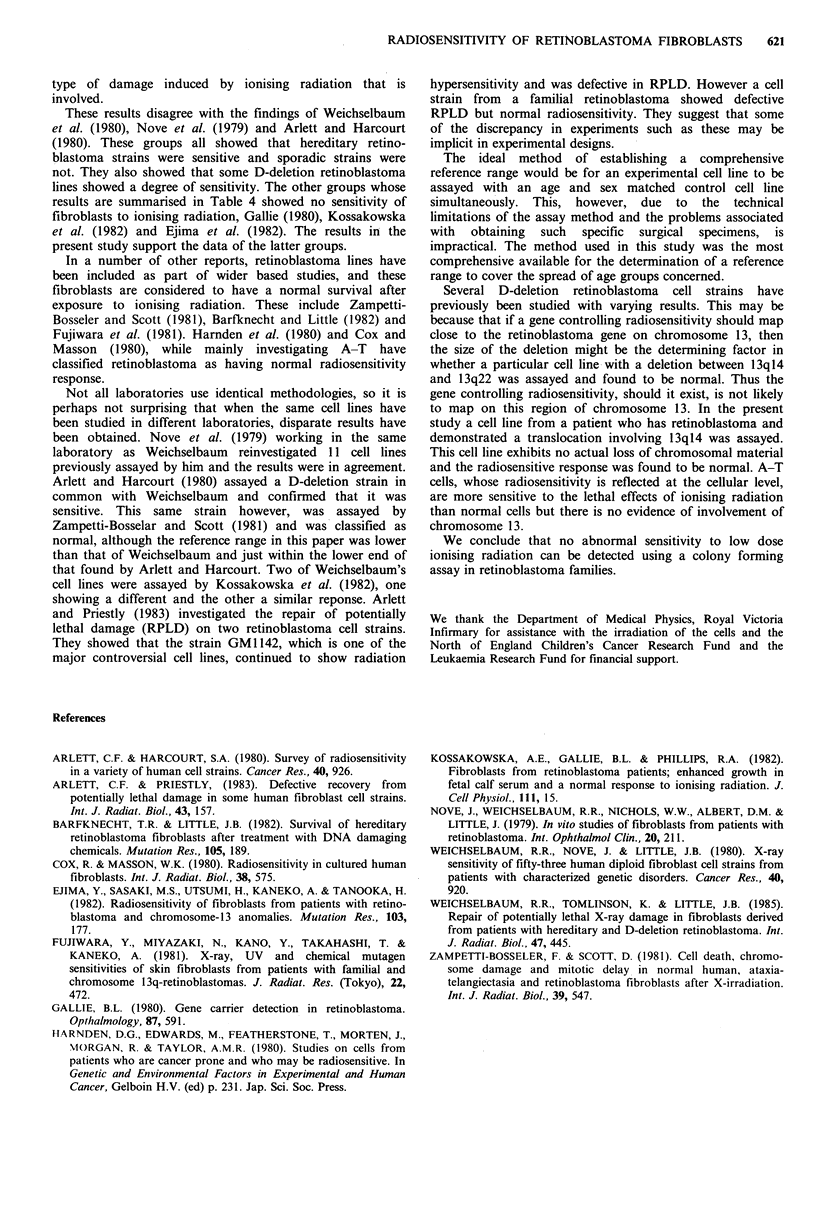


## References

[OCR_00950] Arlett C. F., Harcourt S. A. (1980). Survey of radiosensitivity in a variety of human cell strains.. Cancer Res.

[OCR_00954] Arlett C. F., Priestley A. (1983). Defective recovery from potentially lethal damage in some human fibroblast cell strains.. Int J Radiat Biol Relat Stud Phys Chem Med.

[OCR_00959] Barfknecht T. R., Little J. B. (1982). Survival of hereditary retinoblastoma human skin fibroblasts after treatment with DNA-damaging chemicals.. Mutat Res.

[OCR_00964] Cox R., Masson W. K. (1980). Radiosensitivity in cultured human fibroblasts.. Int J Radiat Biol Relat Stud Phys Chem Med.

[OCR_00968] Ejima Y., Sasaki M. S., Utsumi H., Kaneko A., Tanooka H. (1982). Radiosensitivity of fibroblasts from patients with retinoblastoma and chromosome-13 anomalies.. Mutat Res.

[OCR_00974] Fujiwara Y., Miyazaki N., Kano Y., Takahashi T., Kaneko A. (1981). X-ray, UV and chemical mutagen sensitivities of skin fibroblasts from patients with familial and chromosome 13q- retinoblastomas.. J Radiat Res.

[OCR_00981] Gallie B. L. (1980). Gene carrier detection in retinoblastoma.. Ophthalmology.

[OCR_00992] Kossakowska A. E., Gallie B. L., Phillips R. A. (1982). Fibroblasts from retinoblastoma patients: enhanced growth in fetal calf serum and a normal response to ionizing radiation.. J Cell Physiol.

[OCR_00998] Nove J., Weichselbaum R. R., Nichols W. W., Albert D. M., Little J. B. (1980). In vitro studies of fibroblasts from patients with retinoblastoma.. Int Ophthalmol Clin.

[OCR_01003] Weichselbaum R. R., Nove J., Little J. B. (1980). X-ray sensitivity of fifty-three human diploid fibroblast cell strains from patients with characterized genetic disorders.. Cancer Res.

[OCR_01009] Weichselbaum R. R., Tomkinson K., Little J. B. (1985). Repair of potentially lethal X-ray damage in fibroblasts derived from patients with hereditary and D-deletion retinoblastoma.. Int J Radiat Biol Relat Stud Phys Chem Med.

[OCR_01015] Zampetti-Bosseler F., Scott D. (1981). Cell death, chromosome damage and mitotic delay in normal human, ataxia telangiectasia and retinoblastoma fibroblasts after x-irradiation.. Int J Radiat Biol Relat Stud Phys Chem Med.

